# Characterization of Textile-Insulated Capacitive Biosensors

**DOI:** 10.3390/s17030574

**Published:** 2017-03-12

**Authors:** Charn Loong Ng, Mamun Bin Ibne Reaz

**Affiliations:** Department of Electrical, Electronic and Systems Engineering, Faculty of Engineering and Built Environment, Universiti Kebangsaan Malaysia, Bangi, 43600 Selangor Darul Ehsan, Malaysia; mamun.reaz@gmail.com

**Keywords:** biosensor, capacitive, textile, electromyography

## Abstract

Capacitive biosensors are an emerging technology revolutionizing wearable sensing systems and personal healthcare devices. They are capable of continuously measuring bioelectrical signals from the human body while utilizing textiles as an insulator. Different textile types have their own unique properties that alter skin-electrode capacitance and the performance of capacitive biosensors. This paper aims to identify the best textile insulator to be used with capacitive biosensors by analysing the characteristics of 6 types of common textile materials (cotton, linen, rayon, nylon, polyester, and PVC-textile) while evaluating their impact on the performance of a capacitive biosensor. A textile-insulated capacitive (TEX-C) biosensor was developed and validated on 3 subjects. Experimental results revealed that higher skin-electrode capacitance of a TEX-C biosensor yields a lower noise floor and better signal quality. Natural fabric such as cotton and linen were the two best insulating materials to integrate with a capacitive biosensor. They yielded the lowest noise floor of 2 mV and achieved consistent electromyography (EMG) signals measurements throughout the performance test.

## 1. Introduction

Wearable sensing systems is a trending technology that embeds electronic systems such as biosensors into a garment to provide long-term personal health monitoring capabilities without geographical boundaries [1,2,3]. LifeGuard [4], WEALTHY [5], MagIC [6], and Lifeshirt [7] are good examples of wearable sensing systems that allow users to track their daily physical activities and health condition. Advances in microelectronic technology enable the development of miniaturized biosensors and System-on-Chip (SoC) to address limitations of wearable sensing systems in terms of size, cost, and development time [8]. One of the major hurdles in the adoption of a wearable sensing system is the consistency and accuracy of the bioelectrical signals measurements which are highly dependent on the interfacing methodology between the biosensor’s electrode and the human body.

Generally, there are two types of biosensors, which are wet-contact electrode and non-contact biosensor. Conventional wet-contact electrodes require direct electrical contact between electrode and skin to measure bioelectrical signals emitted from the human body. Usage of wet-contact electrode also require complex skin preparation and a reliance on conductive gel (Ag-AgCl) to reduce the skin-electrode impedance to achieve better bioelectrical signal quality [9]. As a result, it is impractical to integrate the wet-contact electrodes with a wearable sensing system. In contrast, non-contact biosensors such as the capacitive biosensor, operate based on capacitive coupling effect. An air gap, or insulator, is allowed between the electrode and the human body [10]. Neither skin preparations nor conductive gels are required throughout the bioelectrical signal measurement, further simplifying the measurement process. This paper proposes to replace the layer of insulation with suitable textile material to develop a wearable sensor because textile is hypoallergenic, low cost, and easily available in the market.

Every type of textile material has its own electrical and physical properties such as moisture absorption, relative permittivity, and thickness. These properties impact the performance of a capacitive biosensor by affecting noise levels, signal resolution, consistency, and accuracy of the bioelectrical signal measurements. Ueno et al. (2007) developed a mattress with fabric electrodes integrated to measure the electrocardiography (ECG) of a patient [11]. A cotton bedsheet with a thickness of 0.395 mm was used as an insulator between the electrodes and the human body. Although the system was able to measure the bioelectrical signal, but the attenuation of the ECG signals and motion artefacts greatly reduced the signal resolution. Under the European Union (EU) ConText program, Linz et al. (2007) developed the contactless electromyography (EMG) sensors that were embroidered into a textile material for a wearable sensing application [12,13,14]. The sensor’s coupling plate was conductive yarn embroidered onto the textile material. The contactless EMG sensors were able to distinguish the muscle contraction and resting periods. However, removing the common mode noise (50 Hz) and the motion artefacts remained a challenge. Nemati et al. (2012) developed an in-hospital healthcare system with the capacitive ECG sensors that integrated into a cotton T-shirt with a thickness of 0.35 mm [15]. They also investigated the effect of insulating materials on the performance of the capacitive ECG biosensor by comparing the ECG measured by the capacitive biosensors insulated by cotton and wool. The research outcome shows that the ECG measurement results of the capacitive ECG sensor insulated with cotton and wool yielded a relatively low signal amplitude and high common mode noise (60 Hz) in comparison with the dry-contact electrode. The research discussion pointed out that low dielectric constant and the unique electrical properties of wool contributed to the poor signal quality. Yang et al. (2016) developed a dual-electrode capacitive ECG measurement system with cotton fiber as an insulator [16]. However, the system required the implementation of the singular spectrum analysis (SSA) and post signal filtering to obtain higher ECG signal resolution due to the common mode noise and motion artefacts. In summary, research in textile-insulated capacitive (TEX-C) biosensors, with its promising future, has thus far evaluated its usage with only a few textile materials. Since TEX-C biosensors utilized the textile material as a medium of skin-electrode interface, thus, it certainly will affect the noise performance and measurement quality of a TEX-C biosensor. It is important to characterize the effect of textile on the performance of a TEX-C biosensor.

This paper aims to explore and evaluate the capability of six common textile materials to be used as textile insulators for capacitive biosensors. The six textile materials being reviewed in this paper are cotton, linen, rayon, nylon, polyester, and polyvinyl chloride textile (PVC-textile). This paper summarizes the physical and electrical properties of the six textile materials which are commonly used in clothing, bedding, and wearable items. A TEX-C biosensor was developed and presented in this paper. All six textile materials were integrated with the TEX-C biosensor to compare and analyse their characteristics. The performance of these TEX-C biosensors were validated on three subjects, the results of which were benchmarked against the performance of conventional wet-contact electrodes on the same three subjects.

## 2. Methods and Materials

### 2.1. Textile Properties and Characteristic

There are two main categories of textile materials which are natural fabric and synthetic fabric. Natural fabrics are composed of natural materials derived from the fibers of animals and plants. Synthetic fabrics are composed of man-made materials. The natural fabrics used in the experiment are cotton and linen while the synthetic fabrics used in the experiment are rayon, nylon, polyester, and PVC-textile. Table 1 summarizes the physical and electrical properties all six of these textile materials.

Cotton is a natural fiber made from cotton plants. It is commonly used as an insulator for a capacitive biosensor. 100% cotton textile was used in this research study. It is an absorbent and breathable textile which is widely used in clothing [17]. Linen is a natural fiber made from flax plants. It is able to absorb and lose water rapidly, so the humidity of the environment easily affects its electrical characteristics such as the conductivity and dielectric strength [18]. Rayon is made from the regenerated and purified cellulose that is derived from plants and is biodegradable [19,20]. Its elasticity has the advantage of holding the TEX-C biosensor on an uneven body surface [21]. Nylon, also known as polyamide, is commonly used to make raincoats, swimwear, and carpets due to its exceptionally strong abrasion resistance, low moisture absorbency, and elasticity [22]. A nylon type of TEX-C biosensor appears ideal for integration with sportswear to keep track of an athlete’s performance. Polyester is a type of polymer material that is widely use in the apparel and garments. 100% polyester textile was used in this research study. Its high resistance to stretching and shrinking are advantages that ensure the physical characteristic of the insulating material will remain consistent throughout the bioelectrical signals measurement [23]. The PVC-textile being used in this research study is a polyester fabric coated with polyvinyl chloride. The outer layer of plastic polymer is water resistant, non-breathable, and heat sensitive. The advantage of PVC-textile is that humidity does not drastically affect its electrical characteristics.

The thickness of each type of textile was measured using a digimatic caliper series 500 from Mitutoyo with a resolution of 0.01 mm. Each type of textile was cut into a rectangular shape. The thickness of each textile was measured 4 times from 4 different sides of the textile. It is important to note that the thickness measured from 4 different sides of each individual textile consistently yielded the same value. The thickness of each type of textile being used in the experiment were recorded in Table 1. Rayon was the thickest textile in the experiment with the thickness of 0.58 mm. It was followed by nylon, 0.48 mm, and linen, 0.40. PVC-textile, cotton, and polyester were relatively thin, with the thickness of 0.24 mm, 0.23 mm, and 0.16 mm respectively.

The resistance of each textile material was also validated to ensure these materials were neither non-conductive nor contaminated by any conductive substance. Each type of textile material was placed in between two copper plates. A Fluke 117 multimeter was used to measure the impedance between the two copper plates. The Fluke 117 multimeter was set to 0.01 MΩ range with the accuracy of ±1.5%. Table 1 records the resistance values for each type of textile. All the materials have a high resistance value of more than 40.00 MΩ value which is out of the measurement range of the multimeter. The results proved that all the textile materials were insulator.

**Figure 1:**
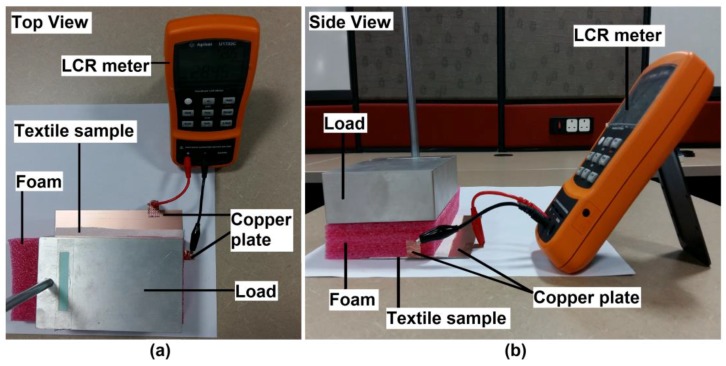
The (**a**) top view and the (**b**) side view of the relative permittivity’s measurement setup.

Textiles normally present a low relative permittivity as they are very porous materials and the presence of air approaches the relative permittivity to 1. However, the textile material, textile density, and manufacturing process do alter the relative permittivity value. Therefore, the relative permittivity value for each sample of textile material was measured using parallel-plate capacitive model [24,25,26,27]. Figure 1 showed the setup of the measurement. Each type of textile material was mezzanine in between two copper plates. The bottom copper plate had the area size of 15,000 mm^2^ while the top copper plate was 875 mm^2^ in size. A foam piece was placed on top of the parallel-plate capacitive structure to keep the top copper plate and the textile material flat. A 1.8 kg load was placed on top of the foam piece to ensure the parallel-plate capacitive structure stay firmly in place. An Agilent U1733C handheld LCR meter was used to measure the capacitance of the parallel-plate capacitive structure at 1 kHz. This LCR meter has resolution of 0.01 pF and accuracy up to ±0.2%. The relative permittivity of the textile material, *ε_r_* was calculated using Equation (1) and recorded in Table 1.

(1) $$\varepsilon_{r}=\frac{C_{m}\;d_{t}}{\varepsilon_{o}\;A_{t}}$$

where

*ε_o_* is the constant value of the vacuum permittivity, 8.854 × 10^−12^ F/m;
*C_m_* is the capacitance measured by the LCR meter;
*A_t_* is the area size of the top copper plate which is 875 mm^2^;
*d_t_* is the thickness of the textile material.

### 2.2. TEX-C Biosensor

**Figure 2:**
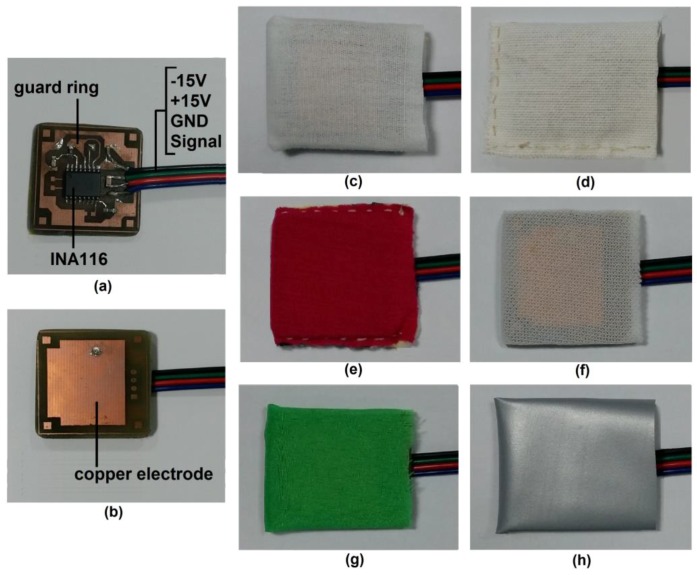
The (**a**) top view and (**b**) bottom view of a capacitive biosensor. The biosensor is insulated by different types of textiles such as (**c**) cotton; (**d**) linen; (**e**) rayon; (**f**) nylon; (**g**) polyester; and (**h**) PVC-textile for experimental purposes.

A TEX-C biosensor was designed and developed based on the parallel-plate capacitive model. It was fabricated on a dual-layer printed circuit board (PCB). One side of the PCB was used as an electrode while the other side contains buffer circuitry as shown in Figure 2. A low input bias current instrumental amplifier, INA 116, is the main component of the buffer circuitry. The copper plate was directly connected to the positive input of INA 116 through a through-hole via. A guard ring was designed to minimize noise coupled to the input of the amplifier. This TEX-C biosensor was powered by +15 V and −15 V to provide a wide range of signal measurement. The copper electrode was placed on the targeted muscle unit with a layer of textile to isolate both of them. The bioelectrical signal was coupled through the textile material and captured by the copper plate. The skin-electrode capacitance, *C_s_*, can be calculated using Equation (2).

(2) $$C_{s}=\frac{\varepsilon_{r}}{d}\times \left(\varepsilon_{0}A\right)$$

where

*ε_o_* is the constant value of the vacuum permittivity, 8.854 × 10^−12^ F/m;
*ε_r_* is the relative permittivity of the textile material;
*A* is the copper electrode’s area size, 510 mm^2^;
*d* is the thickness of textile which control the distance between skin and electrode.

Different types of textile materials have unique *d* and *ε_r_* which control the value of skin-electrode capacitance for a TEX-C biosensor as shown in Equation (2). The distance between the electrode and skin surface was determined by the thickness of the textile material. Although there is a concern that the thickness of a textile might vary due to strain and compression, but these textiles were thin and the value of thickness were measured from 4 corners to avoid discrepancy. Therefore, as long as the TEX-C biosensor is fixed firmly on the human body, the thickness variation throughout the experiment is minimal. The relative permittivity for the different types of textile materials were measured and recorded in Table 1. They were used to calculate the skin-electrode capacitance of the TEX-C biosensors. The area size of the copper electrode remained constant throughout the research study. Table 2 summarizes the skin-electrode capacitance for the six types of TEX-C biosensors. The cotton and PVC-textile type of TEX-C biosensor had a very close skin-electrode capacitance which were 58.96 pF and 58.65 pF. Both cotton and PVC-textile had similar relative permittivity and thickness. Skin-electrode capacitance of linen type of TEX-C biosensor was slightly lower at 45.22 pF. Rayon and polyester types of TEX-C biosensors had mid-range of skin-electrode capacitance at 39.56 pF and 33.24 pF respectively. Nylon type of TEX-C biosensor had the lowest skin-electrode capacitance of 11.49 pF.

### 2.3. Experimental Setup

The environmental temperature, humidity, and subject’s perspiration are factors that might affect the relative permittivity of a textile material. The humidity of the environment and subject’s perspiration would reduce the dielectric strength of the textile with absorbent characteristics and introduce additional substance between the electrode and human body. Thus, it was essential to perform the experiment in an enclosed and controlled environment to minimize the changes of room temperature, humidity, and subject’s perspiration. All the experiments and validations were performed in an air-conditioned electronics laboratory with the room temperature controlled at 23 °C.

The TEX-C biosensor was connected to a data acquisition system which contained a bandpass filter, an analogue-to-digital converter (ADC), a negative feedback electrode, and a host computer. The hardware architecture of a TEX-C data acquisition system is illustrated in Figure 2. The bandpass filter was used to attenuate both high frequency and low frequency noise in the EMG signals. The bandpass filter had a passband frequency range between 10 Hz and 300 Hz. This system had a gain value of 1. The EMG signals were digitized by an ADC with a sampling rate of 600 samples per second. The recorded EMG data was stored in a host computer. The user could monitor and analyse the acquired EMG signals through the host computer. The negative feedback electrode was designed based on driven-right-leg (DRL) methodology for common mode noise cancelation [28].

**Figure 3:**
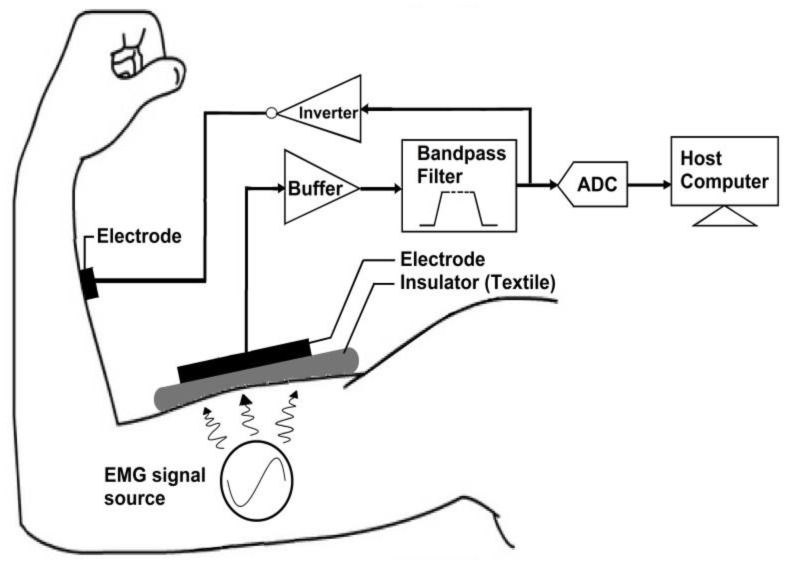
Hardware architecture of the TEX-C data acquisition system.

The validation process of TEX-C biosensor was divided into two sections; which are the biosensors’ characterization and the biosensors’ performance evaluation. The six types of TEX-C biosensors shown in Figure 2 were evaluated on three healthy subjects. All the subjects were not required to undergo skin preparation because the TEX-C biosensor is capable of measuring the bioelectrical signal without galvanic contact from human body. All the subjects’ bicep brachii were checked to ensure that there was no perspiration before the TEX-C biosensor was placed. The TEX-C biosensor was placed on the bicep brachii of the subject as illustrated in Figure 3. Each of the TEX-C biosensors were tested one type at a time. The TEX-C biosensor was held tightly on the bicep brachii of the subjects using a rubber strap. A conventional wet-contact electrode (Ag-AgCl) was placed right beside the TEX-C biosensor to ensure both captured the same EMG signals at the same time for the performance evaluation.

## 3. Results and Discussions

### 3.1. Noise Floor and Characterization

The characterization experiment was performed to analyse the initial characteristics of TEX-C biosensors. The experimental results also served as a baseline value for the performance evaluation. This experiment consisted of two subsections which were the noise floor measurement and the EMG burst signal measurement. EMG signals are weak electrical signals that range from 0.1 mV to 100 mV [29]. The noise floor of the TEX-C biosensor ought to be as low as possible to maximize the SNR.

Firstly, the process of noise floor measurement required the three subjects to keep their bicep brachii at ease so that no EMG signals were generated. Figure 3 shows the average amplitude of noise floor (*V_noise_*) recorded from the three subjects’ bicep brachii using the six types of TEX-C biosensors and the wet-contact electrode. The experimental result showed that the skin-electrode capacitance was inversely proportional to the *V_noise_* value of the TEX-C biosensor. The lower the skin-electrode capacitance, the higher the *V_noise_*. Nylon insulated TEX-C biosensor recorded the highest *V_noise_* which is 32.80 mV while it had the lowest skin-electrode capacitance value of 11.49 pF. Polyester insulated TEX-C biosensor recorded the second highest *V_noise_* which is 4.67 mV with the second lowest skin-electrode capacitance value of 33.24 pF. With the third lowest skin-electrode capacitance of 39.56 pF, rayon insulated TEX-C biosensor recorded the third highest *V_noise_* of 2.79 mV. Cotton insulated TEX-C biosensors had the highest skin-electrode capacitance of 58.96 pF, thus it also recorded the lowest *V_noise_* of 1.65 mV among different types of textile materials. Linen insulated TEX-C biosensors had the second highest skin-electrode capacitance of 45.22 pF, therefore, it recorded the second lowest *V_noise_*, which is 1.99 mV. PVC-textile insulated TEX-C biosensors yielded a slightly different behavior due to its non-porous characteristic as compare to other textile samples. Although PVC-textile insulated TEX-C biosensors had a skin-electrode capacitance value closed to cotton which is 58.65 pF, but it recorded *V_noise_* of 2.52 mV, 0.87 mV higher than cotton insulated TEX-C biosensors. 

Generally, it was expected to observe low amplitude voltage spikes from the output of the TEX-C biosensors which is known as noise floor [30]. The noise floor is commonly contributed by the common mode noise and thermal noise. A high input impedance TEX-C biosensor is highly susceptible to common mode noise such as 50/60 Hz power line noise from an unshielded environment. The voltage of common mode noise, *V_CM_* can be represent by the following Equations (3) and (4). Equation (5) was derived by substituting Equation (2) into Equation (4).

(3) $$V_{CM}=I_{CM}Z_{C}$$

(4) $$V_{CM}=I_{CM}\;\left(\frac{1}{j\omega C_{s}}\right)$$

(5) $$V_{CM}=I_{CM}\;\left(\frac{1}{j\omega \varepsilon_{o}A}\right)\;\left(\frac{d}{\varepsilon_{r}}\right)$$

where

*I_CM_* is the induced current from the human body;
*Z_C_* is the impedance between the skin and the TEX-C biosensor.

**Figure 4:**
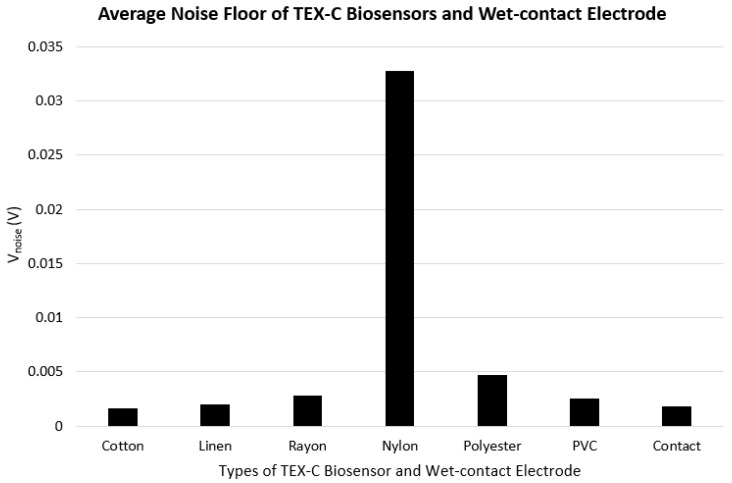
The *V_noise_* recorded on 3 subjects using 6 types of TEX-C biosensors and a wet-contact electrode.

Equation (4) proved that *C_s_* is inversely proportional to the *V_CM_* which was aligned with the results shown in Figure 4. Equation (5) showed that, the parameters affected by the textile material such as *d* and *ε_r_* were contributing factors to the common mode noise. Base on Equation (5), reduce the thickness or increase the relative permittivity of the textile are alternatives to reduce the amplitude of the common mode noise. The *V_CM_* can also be suppressed by implementing a negative feedback between the measurement system and the human body as shown in Figure 3. However, a minimum amount of *V_noise_* was recorded. A 50 Hz/60 Hz notch filter implementation to the measurement system is recommended to further reduce the amplitude of the common mode noise. The electronic system also generated noise such as the thermal noise which is unavoidable. Thermal noise is contributed by the motion of the charge carriers_._ The root-mean-square (RMS) voltage of the thermal noise, *V_T_* can be calculated using the following Equation (6).

(6) $$V_{T}=\sqrt{4k_{B}TR\Delta f}$$

where

*k_B_* is the Boltzmann’s constant, 1.38 × 10^−23^ J/K;
*T* is the temperature of the system in Kelvin;
*R* is the resistance value of a resistor;
∆*f* is the bandwidth which the noise is measured.

**Figure 5:**
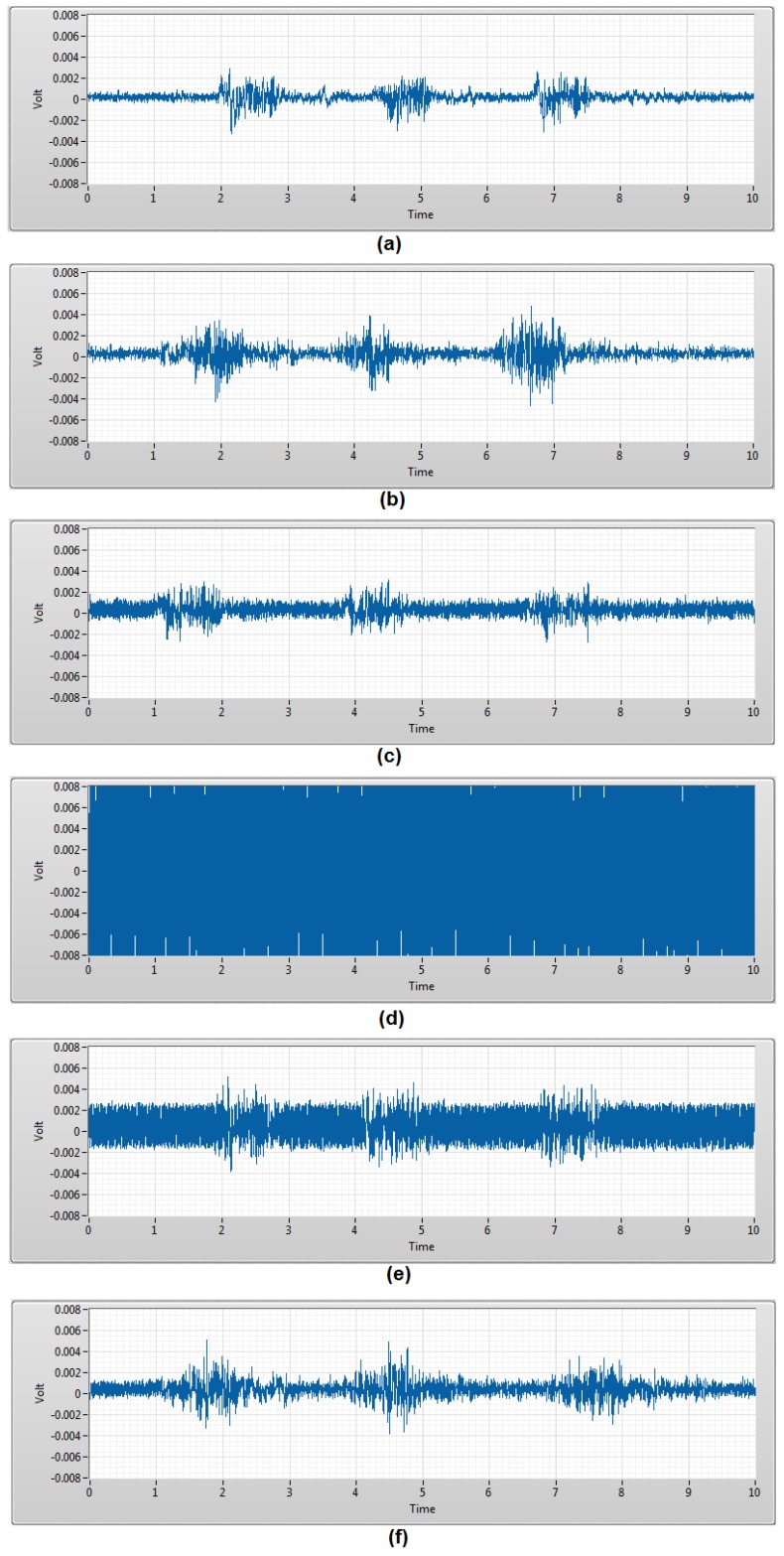
Three EMG burst signals acquired by the 6 types of TEX-C biosensors in 10 s. (**a**) cotton; (**b**) linen; (**c**) rayon; (**d**) nylon; (**e**) polyester; and (**f**) PVC-textile.

Secondly, the EMG burst signal measurement was used to validate the capability of the TEX-C biosensors to acquire the EMG signals from human body. Each subject was required to contract the bicep brachii muscle three times within 10 s to generate 3 EMG burst signals. This experiment was repeated using the six types of TEX-C biosensors on each subject. Figure 5 shows the EMG burst signals acquired from subject A using the different types of TEX-C biosensors. Cotton and linen types of TEX-C biosensor recorded the lowest noise floor so the 3 EMG burst signals developed during the contraction of bicep brachii were clearly observed. Some of the examples of the EMG burst signals were the voltage spikes between 2 s and 3 s in Figure 5a and between 6.25 s and 7.25 s in Figure 5b. The duration of each EMG burst signal was around 1 s. Motion artefacts such as the single voltage spike between 3.5 s and 3.75 s in Figure 5a and the low amplitude of burst signal between 5.35 s and 5.65 in Figure 5b also can be identified. These motion artefacts were mainly coupled from the movement of the muscle near to the targeted muscle unit. Rayon and PVC-textile types of TEX-C biosensor recorded a relatively high noise floor compared to the cotton and linen types of TEX-C biosensor but the 3 EMG burst signals in Figure 5c,f were still visible. The polyester type of TEX-C biosensor recorded the second highest noise floor that ranges between −1.0 mV and 2.5 mV. The 3 EMG burst signals can be hardly observed in Figure 5e. The nylon type of TEX-C biosensor recorded the highest noise floor, which was out of the range of −8 mV to 8 mV. The high noise floor saturating the input buffer and the 3 EMG burst signals were completely unobservable. The *V_noise_* recorded in Figure 4 correlated with the noise floor characteristics shown in Figure 5. The higher the *V_noise_* of the TEX-C biosensor, the lower the signal-to-noise ratio.

Most of the textile samples tested in the experiment had a smooth surface. When the subjects were contracting their bicep brachii, the TEX-C biosensor would tend to have a minimal variation of its position due to the changes in body surface. The friction between the copper electrode and textile material created unwanted electrical charges that translated to the voltage spike in the measurement. As a result, there were multiple voltage spikes before and after the EMG burst signal as shown in Figure 5. Although the current design of the textile material was a tight fit to the TEX-C biosensor, but it is recommended to embroider or glue the textile insulator firmly to the TEX-C biosensor to minimize the friction between the electrode and the textile material. Post-signal processing, feature extraction, and analysis were recommended to filter unwanted signals and analyse the muscle activity.

### 3.2. Performance Evaluation

**Figure 6:**
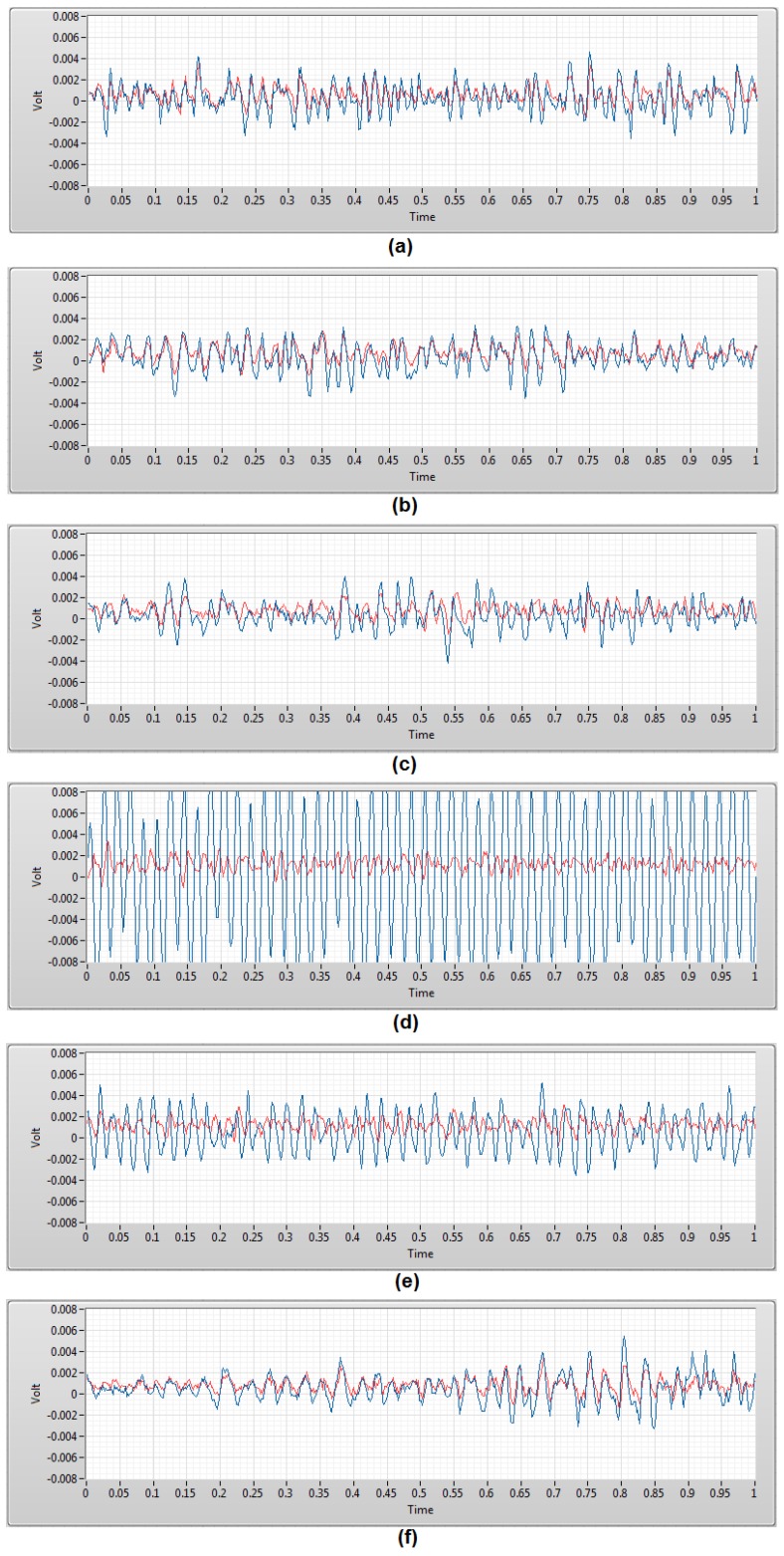
EMG signals were acquired using wet-contact electrode (red) and the 6 types of TEX-C biosensors (blue) in a 1 s duration. The types of TEX-C biosensors are (**a**) cotton; (**b**) linen; (**c**) rayon; (**d**) nylon; (**e**) polyester; and (**f**) PVC-textile.

The characterization test recorded a baseline result of each TEX-C biosensor. The performance evaluation test evaluated the consistency and accuracy of the TEX-C biosensors in EMG signal measurements. The performances of these TEX-C biosensors were benchmarked with the conventional wet-contact electrode (Ag-AgCl) because it is commonly used in clinical applications. Figure 6 shows the EMG signals generated when subject A’s bicep brachii contracted for 1 s. The EMG signals were measured using both TEX-C biosensors and wet-contact electrode.

The EMG signals measured by the cotton and linen types of TEX-C biosensor and wet-contact electrode were similar. These results show that the low *V_noise_* of a TEX-C biosensor is an important factor to capture a high resolution EMG signal. The EMG signals measured by the rayon and PVC-textile types of TEX-C biosensor and wet-contact electrode were slightly mismatched. Rayon and PVC-textile types of TEX-C biosensor measured higher amplitude and shifted the EMG signals in comparison with the EMG signals measured by the wet-contact electrode. The smooth surface of the textile material might cause some minimal movement of the TEX-C biosensor during muscle contraction. As a result, the measurement system was capturing a slightly offset signal in time in comparison with the wet-contact electrode while maintaining the same amplitude. In contrast, the signals acquired by the nylon and polyester types of TEX-C biosensor are completely different from the signals acquired by the wet-contact electrode. The signals shown in Figure 6d,e were 50 Hz periodical sine wave with variation in amplitude. These results showed that the power line noises from the surrounding had saturated the nylon and polyester types of TEX-C biosensor, and no EMG signals can be observed.

**Figure 7:**
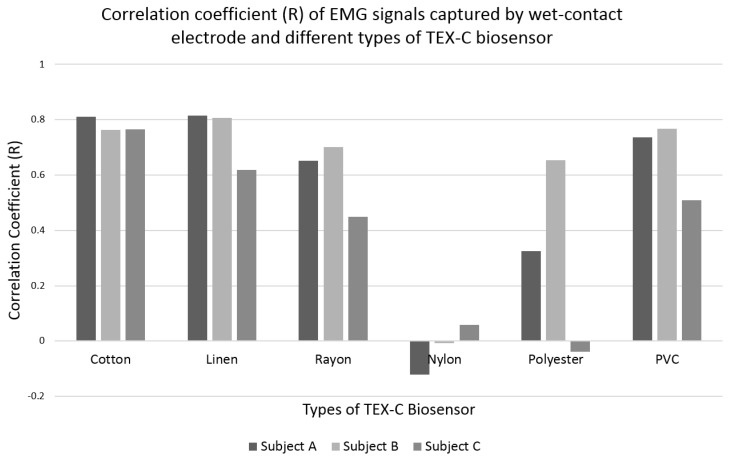
Correlation coefficient results of the EMG signals measured by the wet-contact electrode and the 6 types of TEX-C biosensors among the 3 subjects.

The TEX-C biosensors were tested on three subjects. The correlation results of the EMG signals measured using the TEX-C biosensors and the wet-contact electrode were shown in Figure 7. It was expected that the correlation coefficient value will not achieved 1.0 because both wet-contact electrode and TEX-C biosensor were not placed at the same location but close to each other. The correlation coefficient values of the cotton type of TEX-C biosensor were high and consistent among the three subjects. All three correlation coefficient values were above 0.76. The linen type of TEX-C biosensor also achieved high correlation coefficient values but the results among the three subjects were slightly inconsistent. Subjects A and B recorded correlation coefficient values above 0.80 while subject C had only 0.62. The rayon and PVC-textile types of TEX-C had similar correlation coefficient values among the three subjects. The EMG signals measured from subject C yielded correlation coefficient values of below 0.51 while subjects A and B yielded correlation coefficient values of above 0.65. The rayon and PVC-textile types of TEX-C also had inconsistent measurement results. The correlation coefficient values of the nylon type of TEX-C biosensor was between −0.12 and 0.06 among the three subjects. These results proved that the EMG signals captured by the nylon type of TEX-C biosensor and the wet-contact electrode were completely different. The correlation coefficient values of the polyester type of TEX-C biosensor were relatively low and inconsistent. Subjects A, B and C yielded the correlation coefficient values of 0.32, 0.65 and −0.04.

Overall, the measurement results of natural fabric-insulated TEX-C biosensors recorded lower noise floor and higher correlation values compared to the synthetic fabric. The variation of between the EMG signals’ amplitude and shifted signals measured by natural fabric-insulated TEX-C biosensors were insignificant. The experimental results showed that the performance of different insulators for the TEX-C biosensor was determined by the noise level of each type of sensor. The lower the noise floor, the higher the resolution of the bioelectrical signal measured and better results correlation with conventional wet-contact electrode. The noise level of a TEX-C biosensor is determined by the types of textile insulator because they are the only skin-electrode interface connecting the bioelectrical measurement system to the human body. A high-resolution bioelectrical signal is always preferable to maintain the accuracy and reliability of the measurement result and reduces the complexity of post signal processing.

## 4. Conclusions

Wearable sensing systems have an enormous potential to improve the process of medical treatment and rehabilitation by extending the functionality of smart garments. Different types of textile materials have unique physical and electrical properties that will alter the performance of a TEX-C biosensor. Six types of TEX-C biosensors were evaluated in this paper in terms of noise level, signal resolution, and measurement consistency. The experimental results revealed that every type of textile material has its own unique relative permittivity and thickness that alter the skin-electrode capacitance of a TEX-C biosensor. It is recommended to choose a thin textile material with high relative permittivity as an insulator to maximize the skin-electrode capacitance of a TEX-C biosensor. High skin-electrode capacitance will result in low noise floor and yield a better performance. Natural fabrics such as cotton and linen with high skin-electrode capacitance were recommended textile materials for use as an insulator for the TEX-C biosensor. The EMG signals measured by the cotton type of TEX-C biosensor were consistent, highly correlated with the EMG signals measured by the conventional wet-contact electrode, and yielded the lowest noise floor. The EMG signals measured by the linen type of TEX-C biosensor were slightly inconsistent but correlated with the EMG signals measured by the conventional wet-contact electrode and also yielded a low noise floor. The other TEX-C biosensors insulated by synthetic fabrics such as rayon, nylon, polyester and PVC-textile with low skin-electrode capacitance had relatively high noise floors, which reduced the accuracy and consistency of the EMG signal measurements.

## Figures and Tables

**Table 1:** Properties and characteristics of the textile materials under test.

Textile Types	Category	Thickness (mm)	Resistance (Ω)	Relative Permittivity (*ε_r_*) at 1 kHz	Physical Characteristic
Cotton	Natural	0.23	>40.0 M	3.004	Absorbent, Breathable
Linen	Natural	0.40	>40.0 M	4.007	Absorbent, Breathable
Rayon	Synthetic	0.58	>40.0 M	5.082	Breathable, Elasticity *
Nylon	Synthetic	0.48	>40.0 M	1.222	Breathable, Elasticity *
Polyester	Synthetic	0.16	>40.0 M	1.178	Breathable
PVC-textile	Synthetic	0.24	>40.0 M	3.118	Waterproof, Non-breathable

* Elasticity—the ability of stretch material to return immediately to its original size.

**Table 2:** Parameters to determine the skin-electrode capacitance of the TEX-C biosensors.

Insulators of TEX-C Biosensor	Electrode Surface Area, A (mm^2^)	Relative Permittivity, *ε_r_* at 1 kHz	Thickness, d (mm)	Skin-Electrode Capacitance, *C_s_* (pF) *
Cotton	510	3.004	0.23	58.96
Linen	510	4.007	0.40	45.22
Rayon	510	5.082	0.58	39.56
Nylon	510	1.222	0.48	11.49
Polyester	510	1.178	0.16	33.24
PVC-textile	510	3.118	0.24	58.65

* calculated using Equation (2) with *ε_o_* = 8.854 × 10^−12^ F/m.

## References

[1] Castano L.M., Flatau A.B. (2014). Smart fabric sensors and e-textile technologies: A review. Smart Mater. Struct..

[2] Stoppa M., Chiolerio A. (2014). Wearable Electronics and Smart Textiles: A Critical Review. Sensors.

[3] Mukhopadhyay S.C. (2014). Wearable sensors for human activity monitoring: A review. IEEE Sensors J..

[4] Mundt C.W., Montgomery K.N., Udoh U.E., Barker V.N., Thonier G.C., Tellier A.M., Ricks R.D., Darling R.B., Cagle Y.D., Cabrol N.A. (2005). A multiparameter wearable physiological monitoring system for space and terrestrial applications. IEEE Trans. Inf. Technol. Biomed..

[5] Paradiso R., Loriga G., Taccini N. (2005). A wearable health care system based on knitted integral sensors. IEEE Trans. Inf. Technol. Biomed..

[6] Di Rienzo M., Rizzo F., Parati G., Brambilla G., Ferratini M., Castiglioni P. MagIC system: A new textile-based wearable device for biological signal monitoring applicability in daily life and clinical setting. Proceedings of the 27th Annual International Conference Engineering in Medicine and Biology Society.

[7] Heilman K.J., Porges S.W. (2007). Accuracy of the LifeshirtR (Vivometrics) in the detection of cardiac rhythms. Biol. Psychol..

[8] Pantelopoulos A., Bourbakis N.G. (2010). A survey on wearable sensor-based systems for health monitoring and prognosis. IEEE Trans. Syst. Man Cybern. Part C.

[9] Chi Y.M., Jung T.-P., Cauwenberghs G. (2010). Dry-Contact and noncontact biopotential electrodes: Methodology review. IEEE Rev. Biomed. Eng..

[10] Sun Y., Yu X.B. (2016). Capacitive Biopotential Measurement for Electrophysiological Signal Acquisition: A Review. IEEE Sensors J..

[11] Ueno A., Akabane Y., Kato T., Hoshino H., Kataoka S., Ishiyama Y. (2007). Capacitive sensing of electrocardiographic potential through cloth from the dorsal surface of the body in a supine position: A preliminary study. IEEE Trans. Biomed. Eng..

[12] Linz T., Gourmelon L., Langereis G. Contactless EMG sensors embroidered onto textile. Proceedings of the International Workshop on Wearable and Implantable Body Sensor Networks (BSN 2007).

[13] Gourmelon L., Langereis G. Contactless sensors for surface electromyography. Proceedings of the 28th Annual International Conference of the IEEE Engineering in Medicine and Biology Society: Engineering Revolution in BioMedicine.

[14] Langereis G., de Voogd-Claessen L., Spaepen A., Siplia A., Rotsch C., Linz T. ConText: Contactless sensors for body monitoring incorporated in textiles. Proceedings of the IEEE International Conference on Portable Information Devices.

[15] Nemati E., Deen M.J., Mondal T. (2012). A wireless wearable ECG sensor for long-term application. IEEE Commun. Mag..

[16] Yang B., Yu C., Dong Y. (2016). Capacitively coupled electrocardiogram measuring system and noise reduction by singular spectrum analysis. IEEE Sensors J..

[17] Salvado R., Loss C., Goncalves R., Pinho P. (2012). Textile materials for the design of wearable antennas: A survey. Sensors.

[18] Zilinskas P.J., Lozovski T., Jankauskas V., Jurksus J. (2013). Electrostatic Properties and Characterization of Textile Materials Affected by Ion Flux. Mater. Sci..

[19] Park C.H., Kang Y.K., Im S.S. (2004). Biodegradability of cellulose fabrics. J. Appl. Polym. Sci..

[20] Buschle-Diller G., Zeronian S.H., Pan N., Yoon M.Y. (1994). Enzymatic hydrolysis of cotton, linen, ramie, and viscose rayon fabric. Texile Res. J..

[21] Basu S. Tensile Deformation of Fiber Used in Textile Industry. http://cp.literature.agilent.com/litweb/pdf/5991–0274EN.pdf.

[22] Zhang W., Wu C.W., Tan Y.Y., Silva S.R.P. (2012). Functionalisation of nylon with carbon nanotubes to make thermally stable fabric and wearable capacitor. Micro Nano Lett..

[23] Sankaralingam S., Gupta B. (2010). Determination of Dielectric Constant of Fabric Materials and Their Use as Substrates for Design and Development of Antennas for Wearable Applications. IEEE Trans. Instrum. Meas..

[24] Marjanovic M., Paunovic V., Prijic Z., Prijic A., Dankovic D., Mitic V. One the measurement methods for dielectric constant determination in Nb/BaTiO_3_ ceramics. Proceedings of the X International Symposium on Industrial Electrinics (INDEL).

[25] Jilani M.T., Rehman M.Z., Khan A.M., Khan M.T., Ali S.M. (2012). A brief review of measuring techniques for characterization of dielectric materials. Int. J. Inf. Technol. Electr. Eng..

[26] Tereshchenko O.V., Buesink F.J.K., Leferink F.B.J. An overview of the techniques for measuring the dielectric properties of materials. Proceedings of the General Assembly and Scientific Symposium.

[27] Grove T.T., Masters M.F., Miers R.E. (2005). Determining dielectric constants using a parallel plate capacitor. Am. J. Phys..

[28] Kim K.K., Lim Y.K., Park K.S. Common mode noise cancellation for electrically non-contact ECG measurement system on a chair. Proceedings of the 2005 IEEE Engineering in Medicine and Biology 27th Annual Conference.

[29] Beck T.W., Housh T.J., Cramer J.T., Weir J.P., Coburn J.W., Malek M.H. (2006). Electromyographic instantaneous amplitude and instantaneous mean power frequency patterns across a range of motion during a concentric isokinetic muscle action of the biceps brachii. J. Electromyogr. Kinesiol..

[30] Huigen E., Peper A., Grimbergen C.A. (2002). Investigation into the origin of the noise of surface electrodes. Med. Biol. Eng. Comput..

